# Look Upstream: Measurement for Innovation on the Upper Rio Negro of the Amazon Basin

**DOI:** 10.3389/fped.2020.567257

**Published:** 2021-01-27

**Authors:** Kurt Shaw, Rita de Cácia Oenning da Silva

**Affiliations:** ^1^Usina da Imaginação, Florianópolis, Brazil; ^2^Universidade Federal de Santa Catarina, Florianópolis, Brazil

**Keywords:** indigenous children and families, early childhood development, multilingualism, Amazonia (Brazil), video coaching

## Abstract

The growth of the randomized controlled trial (RCT) as the “gold standard” for evaluation has justly been praised as an advance in the professionalization of social programs and projects, an “adoption of science” - in the words of the Lancet. None the less, the emphasis on the RCT biases funding for projects that distribute private goods and which focus on “low hanging fruit” in health, nutrition, and sanitation, simply because those areas lend themselves to the sort of measurement that works with RCTs. As a result, many project developers in the government and NGO sectors lament that a hegemonic focus on RCTs impedes creativity or new models that challenge traditional paradigms. This case study of CanalCanoa, a community video coaching project for indigenous parents of young children in the Rio Negro region of the Amazon Basin, offers techniques to measure for innovation. Instead of developing a new RCT for an extremely diverse population (27 ethnic groups) where traditional childcare methods are in historical flux because of urbanization, CanalCanoa measured variables shown by previous RCTs to be causally connected with positive development results. By researching the impact of the intervention on nutrition, language (multilingualism, use of traditional songs and stories), and social network expansion, CanalCanoa measured upstream indicators, thus mixing scientific rigor with an opportunity for innovation and providing important insight and reform of a theory of change.

## Introduction

Indigenous children of the Amazon enjoy a wealth of resources for their intellectual, physical, and social development: the liberty to explore the jungle and experience science-as-curiosity, the joy of collective play, the chance to learn side by side with parents as they paddle, hunt, and garden (1). Neuroscientific studies show these elements as essential to brain development in early childhood. At the same time, provision of services is extremely difficult in a region where parents living in villages must paddle as many as 15 days in order to reach a hospital or pharmacy and the impact of the modern world has made material conditions extremely difficult. Local political leaders, knowing that their funding from the federal government depends upon improvements in health statistics, threaten retaliation and even death to nurses and doctors who report accurate data. For this reason, we see wild fluctuations in data, with child mortality at 15 one year and 150 the next: without objective data, project planning and evaluation are almost impossible (2).

Innovation in service delivery is essential to addressing the diversity and complexity of needs of 27 ethnic groups speaking 22 different languages. However, small sample sizes, extraordinary difficulty of access, and the lack of accepted, standardized metrics for impact results challenge the capacity to prove any concept intervention though a randomized, controlled trial. With CanalCanoa^1^, indigenous people document their songs, stories, and childrearing practices on digital media and then a team of indigenous educators showed the resulting films to dozens of small groups, to stimulate discussion, reflection, and cultural action for early childhood. In order to evaluate the impact of this initiative, we decided to “look upstream,” both physically and metaphorically, using the way local people think about the flow of water to develop and evaluate project results. CanalCanoa looked upstream toward the traditional villages above the town of São Gabriel in Brazil to help indigenous people to document their songs, stories, and childrearing practices in some 53 movies; a team of indigenous educators then showed the resulting films to more than 30 groups of 10–25 parents as a way to stimulate discussion, reflection, and cultural action for early childhood.

The evaluation system also “looks upstream”: instead of directly measuring impact on children, CanalCanoa measured variables – intermediate outcomes in parlance of RCTs – shown by previous RCTs to be causally connected with positive development results. In this way, the evidence collected through the process of the project could become part of a feedback loop to address specific issues; project adaptations could be informed by evidence in dialogue with previous research. By researching the impact of the intervention on nutrition practices, language use (multilingualism, use of traditional songs and stories), and social network expansion, CanalCanoa measured upstream indicators, thus mixing scientific rigor with an opportunity to test real innovation.

## The Challenges of the RCT

Over the last decades, the randomized controlled trial (RCT) has come to occupy a central role in the development and funding of interventions in many areas of health and social services. Comparing before and after the year 2000, we see a 30-fold increase in the production of RCTs (3). “The World Bank's Independent Evaluation Group reports that over 80% of the [evaluations] starting in 2007–10 used randomization, as compared to 57% in 2005–06 and only 19% in prior years”. This growth, which led to the awarding of the 2019 Nobel Prize in economics to the “randomistas” Abhijit Banerjee, Esther Duflo, and Michael Kremer for their experimental approach to alleviating global poverty, solidified this model as the “gold standard” in development economics. Without a doubt, this movement has added important scientific rigor and improved effectiveness of many social projects.

In spite of this positive impact, critics have pointed to several important negative impacts of the hierarchy of evaluation that puts RCTs at the top.

“Randomization tends to be better suited to relatively simple programs, with clearly identified participants and non-participants, relatively short time horizons, and with little scope for the costs or benefits to spillover to the group of non-participants. In short, the tool is better suited to private goods, which are easy to assign across individual households, but are less able to handle public goods with benefits shared across many people.” ^2^.

The methodological debate in other scientific literature can offer interesting insights into the strengths and weaknesses of the RCT. Volpp et al. have compared innovation in medical research to what happens in software development and technology; examining various meta-analyses of development in both areas, they showed that the RCT model is “best suited for incremental innovations in low uncertainty environments.” [(4), p 4, see also (5)] When the basic intervention paradigm has been established, RCT evaluations can fine tune and perfect the model in useful ways; they can also measure the effects of different well-established models. RCTs do not work as well, however, when the goal is a radical transformation of a model or adaptation to a completely new context.

The context of the Upper Rio Negro plays against the ways that RCTs work best: first, diversity begets diverse responses. People in the region speak 22 different languages. Though many riverine communities share similar ideas of childhood and health, Yanomamis and Nadehups see pregnancy, childbirth, and child development in a radically different way from their indigenous peers. Families may live a hunter-gather lifestyle, one based on fishing and subsistence farming, or in urban slums. The needs of children and families in all of these groups demand a complex and flexible intervention, exactly the kind that RCTs find it difficult to measure. Secondly, just as the small rivers that support remote villages flow into the huge Rio Negro, the close-knit social networks that define indigenous life mean that project benefits will spill over into any control group within the same ethnic group: families share ideas, childcare, and even goods that might seem private. This spillover makes randomization impossible and a control group non-existent. In the heterogeneous context of the upper Rio Negro, an intervention must be flexible to respond to many different needs and demands—and the system of evaluation must also have that flexibility.

At various conferences in which we have participated over the last several years, conversations between academics and program designers have turned to the topic of innovation. Though respecting the need for rigorous evaluations—and grateful for the insights that RCTs provide in this field—we have heard complaints that this model can be a straight-jacket, limiting innovation and creativity. Program designers simply foreclose certain options before they even could come to light, because they understand that such a model would be difficult or impossible to evaluate with an RCT. Though we have been unable to find published articles analyzing this problem, the universality of the complaint among practitioners and even academic experts suggests that the concern is wide-spread. In fact, part of the motivation for this article came from an American academic who asked, wide-eyed, “How did they even convince a funder to look at something as radically different as CanalCanoa?”

Though local leaders and intellectuals may not understand the details of randomization, they strongly critique the interventions used by the Brazilian government. As one Baniwa leader told us, “A 4 year old here can climb a 15 m tall açaí palm, swim across the river, paddle a canoe, distinguish 10 types of manioc, and probably speak four languages. And some bureaucrat from Brasília thinks he can tell me how to define a healthy kid?” Who, then, has the right to establish the metrics and milestones for childhood development?

Perhaps even more significant, indigenous philosophy on the Amazon believes it deeply unethical to see anything else as an object. For them, every other (even trees and rocks and animals) has its own perspective, its wants and desires. Indigenous epistemology does not try to objectively understand the other, but to see through the eyes of the other (6), revealing a world of knowledge that would have been foreclosed by a rigid, randomized controlled trial. CanalCanoa attempted to integrate this epistemology, open to the unexpected and to the productive force of errors and mistakes, in addition to the scientific rigor of contemporary evaluations.

## CanalCanoa: Up the River

Staring in 2012, in collaboration with indigenous leaders, midwives, shamans, educators, and public policy professionals, the CanalCanoa team first scripted and then filmed hundreds of hours of interviews and supporting images, showing and explaining traditional child-rearing practices of indigenous groups on the upper Rio Negro. Though oriented by the questions of the contemporary science of early childhood development, the seven films that emerged from this cinematic research follow the logic established by these local experts. The project emerged from the basic premise that indigenous communities in the Amazon have excellent and time-tested practices of child development, already adapted for the extreme context of the jungle. Instead of imposing western methods of child development, CanalCanoa proposed to support parents as they reflect on and adapt these traditional techniques to the exigencies of modernity. The idea of “video-coaching” (7) has gained important currency in some childhood development circles as a way to create positive feedback loops for parents: CanalCanoa serves as a kind of cultural video coaching, in which larger communities and ethnic groups can look at themselves in the mirror of the camera and movie screen to evaluate and adapt their own child-rearing.

CanalCanoa originally planned to form 20 neighborhood groups of 10 families each in urban neighborhoods of São Gabriel and in the semi-rural villages that surround it; after 12 months of work, however, additional community groups began to request participation in the project, expanding in the intervention to more than 40 organizations and villages in the region. Each group participated in the same series of workshops, generally seven 2-h sessions over 2 months, though community schedules demanded some flexibility. CanalCanoa educators, a team of five local indigenous intellectuals from the largest ethnic groups in the region (Tukano, Tariano, Desana, Baniwa, and Baré), organized the sessions and served as popular educators and discussion leaders. The *ajuris de conhecimento* (roughly “Knowledge Barn-raising sessions”)^3^ were held in homes, community centers, or *malocas* (great-houses), and as participants arrived, they watched a series of shorts made by indigenous children with CanalCanoa — cartoons based on local legends and stories, documentaries showing how to make toys, how to prepare açaí or tapioca, or how to make different arts and crafts. After the group had gathered, everyone watched one of seven films, shown in seven consecutive sessions, on early childhood development, and then spent more than an hour discussing the ideas and images and relating it to their own lives. Though the word “intervention” is more commonly used in the literature, the project really created a space for interactions, re-closing feedback loops (between parent and child, between parents and extended family/community) that had been short circuited by urbanization and modernization. The *ajuris* were also themselves part of the research and evaluation protocol, because participants suggested new themes to include in the films, suggested other experts to interview, and evaluated the changes in their own lives as they occurred. The project quickly adapted to these new inputs.

The workshops followed the logic of traditional indigenous festivals, taking some elements from the *dabucuri* — a traditional ritual in the region that formalizes the exchange of goods and ideas with new visitors and other cultures (8) — while using aspects from other social encounters as well. The popular educators organized traditional meals for all participants — generally *mujeca* or *quinhampira* (peppery fish stews), açaí with *farinha* or tapioca, or other local foods — to take advantage of the comensality that both anthropologists and local leaders cite as so important in the region (9). This dialogue over the meal and after the film emerged from Paulo Freire's ideas of popular education (10): they are not didactic, but dialogic, intending to evoke the knowledge of the participants and not to impose new learning.

In the cultures of the Rio Negro, small children accompany their parents everywhere, and these workshops were not different. During the meal and the event, children interacted with their parents, siblings, and other adults in diverse ways, and these interactions served as a resource for the discussions. Local experts — midwives, various types of shamans, community health agents, teachers, elders, community leaders — also participated in each workshop, with an enthusiasm beyond what planners has expected. Their presence not only offered depth to the discussion, it also served as a seed for extending these ideas and practices in even larger social networks.

In the language developed by the Measurement for Change initiative, the entire intervention was *inclusive*: the community contributed to the development of the project, benefited directly from learnings, decided on indicators, provided data, and in the end had access to all elements of the data and conclusions. Some 1,186 adults and 1,148 children participated in the project, with an additional 44,000 indirect beneficiaries through network effects, shared films, and cultural impact. This research emerges from semi-structured interviews with participants in 28 of the 29^4^ *Ajuris de Conhecimento;* in some cases, we spoke with individuals who had participated, but other times the whole community requested to be part of the evaluation. Because of the community orientation of the culture of the Upper Rio Negro, we quantified changes by group, and not by individual.

## Discussion of Upstream Indicators

Though CanalCanoa proposed to promote early childhood development in the communities of the upper Rio Negro, it did not have access to the advanced brain scanning devices that could show direct changes in cerebral development. In addition, there is little previous research to provide a baseline for “normal” childhood development in the region, other studies have shown the regular falsification of official data (2), and the tightly interconnected social networks make spillover from the intervention inevitable.

Taking these challenges into consideration, CanalCanoa focused its evaluation efforts on identifying and measuring locally meaningful upstream indicators known to be associated with better results in early childhood development:

1) Intellectual stimulation through language, song, and contact with diverse natural and cultural environments,
2) Appropriate, diverse nutrition and access to health care,
3) Supportive relationships with adults and older children that reduce toxic stress. [(11), pp. 10–18].

### Linguistic and Cognitive Development

Programs working with small children generally test cognition through written, drawn, or oral assessments, but many observers have criticized these methods as biased toward children from western, industrialized cultures, with high levels of formal education [(12, 13), among many others]. During the research to develop the CanalCanoa model, parents and local experts along the Rio Negro commented many times that school psychologists or social workers would evaluate their children using tools developed for urban, non-indigenous children. “They showed a traffic light and asked my boy which color meant stop. How is he possibly supposed to know that?” one mother commented.

The upper Rio Negro enjoys extraordinary linguistic wealth, with 27 ethnic groups speaking 22 different languages (14) divided into several different language families: Tukano, Aruwak, Nadehup (Maku), Yanomami^5^, and Tupi-Guarani. Kinship rules of the riverine groups [Tukano, Aruwak, and some Baré (who are ethnically Arawak but who speak Nheengatu, a form of Tupi-Guarani)] demand intermarriage with groups that speak a language different from one's own mother tongue. As a result, children traditionally would grow up with mothers and fathers who spoke different languages, enabling them to speak at least two, and often four or five, languages. This linguistic context is immensely important for neurological development:

Growing evidence shows that early bilingualism can provide children with benefits that go beyond knowing more than one language. Research has shown for some time that bilingual children typically develop certain types of cognitive flexibility and metalinguistic awareness earlier and better than their monolingual peers (15–18)^6^.

Baniwa and Tukano parents are particularly proud of their children's capacity to control impulses and to govern themselves; interestingly, studies have also shown than bilingualism improves executive function in early childhood development (19, 20). However, as families move from villages into the city — and as village children integrate into the school system — Portuguese has become the dominant (and sometimes only) language. Among children and teenagers who grew up in the urban and semi-rural areas of São Gabriel da Cachoeira, today few speak native languages — though many continue to understand them.

Participants in the *ajuris de conhecimento* report a large impact on language acquisition and linguistic development. Ninety one percent of interviewed participants report that they changed the way they interacted linguistically with their children or grandchildren after the *ajuris*. In 77% of the *ajuris*, participants (or, in the case of communities, the whole village) began to speak more with their small children in native languages. In 55% of the groups, participants began to tell more stories to their children after the *ajuris*, and in 36% of the groups, parents and grandparents sang more to their children. It is also notable that after many of the *ajuris*, older children began to sing and tell stories to babies and request that parents tell stories to them and their younger siblings.

Without developing new cognitive tests or using brain-scanning equipment inaccessible in the region, measurement of multi-lingual education and of the use of songs and stories serves as useful stand-in for intellectual development among indigenous children. The upstream indicator supplied scientific rigor while at the same time allowing for innovation in project development.

### Nutrition and Physical Development

Physical development over the course of childhood was an area where local traditions and government measurement did not see eye to eye. While official milestones of growth track weight and height against age, local experts insisted on radically different metrics to evaluate if a child was growing up well^7^. For them, being able to climb a tree or swim in the river was much more important than height or weight, and they preferred to evaluate with the general happiness and well-being of babies and children. Indigenous experts evaluate activity, not the static body. Government health professionals also recognize that healthy indigenous children can stray far from the national average, due to both genetic and environmental factors^8^ Baniwa and Hup'dah children are simply smaller than their peers São Paulo or Rio de Janeiro; to be small and strong is essential to survival and success in the environment in which they live.

Instead of developing new standards and norms to measure physical development in each indigenous group, CanalCanoa decided to evaluate results based on several local metrics, on the way that parents learned to create a therapeutic itinerary in an urban context, and at how parents of young children feed their children. In this paper we look particularly at nutrition, because both local and national intellectuals agree on definitions of healthy food.

Indigenous people living in the town of São Gabriel da Cachoeira have moved very quickly from a subsistence to a cash economy. As children, they lived in widely distributed villages along the river, where there were plenty of fish for the regional population and where fields for manioc were available — if difficult to cut from the jungle. In town, they have access to cash from work and from the *bolsa fam*í*lia* welfare benefit, but the river near São Gabriel simply cannot provide fish for the whole population of the town, and land for farming is bought and sold at a price far above that what a recent migrant from the jungle can pay. In these circumstances, many families in São Gabriel make the same economic calculations as poor people the world over: how can they get the most calories per dollar? In the best of cases, they chose rice, beans, and frozen chicken, all shipped up the river from Belém or Manaus. Sadly, some families are left to choose the local version of chips, Cheetos, soda pop, and other forms of junk food. Both during the ajuris and in interviews that preceded and followed them, mothers and fathers explained that they would prefer a healthier, more local and traditional alternative. They simply can't afford it.

The evaluation discovered that the *ajuris* served not so much to raise awareness of the need for healthy food, but as brainstorming sessions to find new food sources. In 42% of the urban *ajuris*, participants were inspired to plant food at home, using the small yards of urban houses as they had used the *roças* (manioc fields) in the countryside. The most common plantings were herbs, bananas, pineapple, manioc, and palm fruits like açaí, *tucumã*, and *pupunha*. In other cases, participants in the *ajuris* used the discussion sessions as opportunities for learning from and collaborating with other families: in one group, a mother with extra land in her *roça* outside the city offered it to other mothers; in another group, fathers decided to buy fertile eggs to raise their own chickens. In perhaps the most interesting case — an urban Baré community on the edge of the river — three fathers decided to return to fishing, a practice they had abandoned since coming to the city. We saw these changes in more than 50% of urban *ajuris*.

One story illustrates how this change functions: in the neighborhood of Fortaleza, one participant had an extensive urban garden. During the conversations after the film on nutrition, she explained how she planted, composted, and shared her fruits and vegetables. This experience motivated other women to do the same thing: almost all of the other participants in the group planted their own gardens after the *ajuris*. This anecdote allows us to see that it is not so much the film that inspired changes but the platform that the *ajuris* provide for successful mothers and grandmothers to share their techniques with others. The agent of social change in the process is not the educator, but the other participants.

By looking at upstream indicators of nutrition, CanalCanoa not only discovered that the intervention model improved the fundamental input for healthy physical development (diet), it also explained how the structure of the project encouraged urban indigenous women to teach and support each other. The model of evaluation validated and adapted the model, while at the same time elaborating the theory of change.

### Social Networks to Support Babies

An extensive literature on early childhood development has shown two elements to be essential for healthy physical and mental growth: an extended social network to support babies and their mothers during the challenging first months of a baby's life [(11), p.10; (20–22)], and lowered levels of toxic stress in a baby's immediate environment (23–25).

“Excessive and/or prolonged activation of the stress response systems can disrupt the development of brain architecture and other developing organs. This cumulative toll increases the risk for stress-related disease and cognitive impairment, including heart disease, diabetes, substance abuse, and depression, well into the adult years. Research also indicates that supportive, responsive relationships with caring adults as early in life as possible can prevent or reverse the damaging effects of a toxic stress response” [(11), p. 14].

Sadly, the transition from village to town makes both of these issues difficult for indigenous parents of young children in São Gabriel. Migration breaks up social and kinship networks, while the filthy, loud, and unsafe environment of the slums of São Gabriel creates high levels of toxic stress for babies and young parents who do not have the tools to deal with it. The development or strengthening of social networks for early childhood in urban and semi-rural spaces was one of the most interesting results of CanalCanoa.

Every single interviewed participant cited improved social relations as one of the most important impacts of the *ajuris*. Older participants (grandparents, midwives, community leaders, shamans) affirmed that after seven sessions of conversation with young mothers and fathers — as well as, in some cases, adolescents who had not yet had children — they felt more respected: that their knowledge was valued and that young people saw them as important in the community. The youngest participants in the ajuris all cited the importance of learning to respect — and *show* respect — for their grandparents and parents. Sixty three percent of the interviews cited increased respect for the knowledge and persons of elders as an important result. Fifty nine percent of interviews cited more respect for and better dialogue with young people as a concrete outcome. As a consequence, children had better support networks to help them to resist and elaborate toxic stress, which can counterbalance this problem and build resilience for the future (26).

The strengthening of the social fabric that supports babies and their mothers illustrates several important issues around measurement for change or measurement for innovation. Original research plans did not include social network analysis, but at the end of conversations after the conclusions of the *ajuris*, both researchers and indigenous educators asked if there was anything else that the respondents wanted to mention. Without exception, interviewed participants spontaneously mentioned some form of social network strengthening as one of the most important results of the ajuris, especially the intergenerational issues mentioned above, but also increased trust for other ethnic groups and improved relations with neighbors in urban contexts.

Why were the films and ajuris so successful in strengthening social networks? In almost all of the traditional villages of the Rio Negro, communities begin the day with a community breakfast in the great-house (*maloca*). At 6 AM, everyone who lives in the villages brings *xibé* (manioc porridge), fruits, beiju (manioc tortillas), and soup made of fish, game, and peppers to the great house for a collective problem-solving session. The community plans collective work (cleaning, construction), discusses and votes on how to relate to actors outside the village (government, army, health care providers, the church, other villages, anthropologists), and mediates any conflicts in the community (land use, fishing rights, personal conflict).

When families move from the villages to towns like São Gabriel, they lose this tradition of the daily community meeting. Most move onto streets where they know few other people; cities have few traditions of collective labor; there are few or no *malocas* to host the meetings; and relations to government actors are now mediated through individuals or nuclear families, not through communities.

In hindsight, we could see that the *ajuris de conhecimento* stood in as un urban substitute for the community morning meeting, and that the tradition of this meeting imbued the *ajuris* with sense and purpose. In the *maloca*, people from the community came together to solve problems collectively, and the participants in the intervention framed the *ajuris* in the same way. Families faced problems in the city; in their conversations over *quinhampira* and açaí, they could work to solved these problems. Instead of placing the *ajuris* in the frame of education, the indigenous participants saw them as a productive, child- and woman- centered adaptation of the morning village meeting.

This reflection also points to the importance of local participation in project design. Indigenous intellectuals helped to structure and script the *content* of the films that catalyzed the ajuris, but they also collaborated to create the *form* of the intervention. During long discussions about how to implement the project, a Tukano intellectual and a Baniwa indigenous leader working with the CanalCanoa team repeated insisted on the social and community aspect of the work and suggested the name *ajuri de conhecimento* as the frame for CanalCanoa, instead of the more education-based nomenclature that others had suggested. As André Hipattairi Baniwa told us, “An *ajuri* is collective, and about effort and work, not simply reflection.” The indigenous leaders knew much better than an outsider what form of structure would match with the local world-view; what took us months of research and reflection to understand, they knew intuitively.

The strengthening of social networks also shows the importance of plowing evaluation results back into the intervention quickly. The community aspects of CanalCanoa were expensive and challenging for both logistical and accounting reasons. Supply and demand make both fish and açaí very expensive in São Gabriel, a constant complaint of immigrants from the villages. Urban kitchens are seldom prepared to serve the 30–50 people who would often come for an ajuri. And local fishermen, farmers, and cooks were not able to produce the formal, government-stamped receipts demanded by Brazilian accounting standards. For these reasons, project directors felt pressure to redirect resources. However, the results of the evaluation not only justified the investment, but also encouraged directors to budget even more resources for the community aspect of the intervention.

This result emerged spontaneously from open conversations with participants in the ajuris, because of the open-ended and qualitative elements of the evaluation, and because of the trust that participants felt in the indigenous group leaders and in the research team. Had the evaluation model been a rigid RCT, we would not have perceived this impact, because we did not even know the right questions to ask.

This reflection clarifies that the success of CanalCanoa depends on the fit between the intervention model and the local culture: for this reason, we should have serious doubts about scaling and replicability in cultures where community problem-solving meetings do not play such a central role. However, just as we saw that the *form* of the community problem-solving ajuri was as important as the *content* of the films that catalyzed the meetings, we can say that the *practice* of project design in collaboration with the local community is as important as the *model* that emerges from it. If there is a lesson from CanalCanoa for other projects wishing to innovate, it is not to copy the *ajuri* model, but to develop unique local models together with local intellectuals, harkening to their knowledge and intuition.

### Conclusions of Analysis and Discussion

Organizers hypothesized that participation in the *ajuris de conhecimento* would increase knowledge of traditional indigenous practices and awareness of the importance of these practices for the development of their children. The impact extended far beyond this hypothesis, including not only better attitude and awareness but also concrete changes in practice for almost every group that participated. Research found that even before the beginning of the first session of the *ajuri*, parents and grandparents understood the importance of traditional nutrition and health care, valued language, story, and linguistic interaction, and knew that intergenerational care networks were important for children. The problem was not awareness, but the capacity to do what they thought right in the new context of the city. By looking at upstream indicators instead of administering a rigid RCT, the evaluation not only showed that CanalCanoa had been successful, but it explained *why* it had worked, clarifying the theory of change.

The feedback loop quickly plowed measurement and evaluation back into the intervention model. In this way, the program could adapt ecologically, emphasizing the areas that participants and local leaders found most effective and editing out less useful elements. This measurement for internal change directed attention to the social agency of the community and emphasized the films' role as a catalyst for local encounters where young parents could collaborate with other community members to solve problems and think through the challenges oof modernity. This evaluation teaches that future scaling or adaptation of CanalCanoa would have to use this catalyst function as the center of the intervention.

## Conclusion

Ludwig Wittgenstein (27) insisted that philosophers made a mistake by looking for monocausal explanations for complex phenomena. Thinkers, he insisted, need a large toolbox. Experience and education then teach us which tool is appropriate for which task. Instead of conceiving of the randomized controlled trial as the “gold standard” of program development and evaluation, perhaps it is best to see it as one powerful tool among others.

Thomas Kuhn's analysis of scientific change (28) offers an interesting insight into this process. Kuhn famously argued that most of the time, scientific discovery advances in small steps, refining and perfecting the data inside a certain theory or paradigm. However, as new data stretch the paradigm, scientists must develop more and more exceptions to the theory, until finally some observers see the previous paradigm as contradictory or inviable. With this recognition, a new theory emerges and the data slide into the framework of that theory more easily than they did the previous. Kuhn famously defined this revolution as a paradigm shift.

We would argue that the RCT serves brilliantly to refine and perfect knowledge *within a paradigm*—in this case, a paradigm of service provision instead of scientific knowledge. It also can provide the details that will someday serve to shift that paradigm toward something else. However, this model of evaluation is not conducive to paradigm shifts; it is complementary and necessary, but not sufficient.

Though many project developers feel constrained by the RCT model, see it as foreclosing possibilities for interesting innovations before they can even be fully thought through, CanalCanoa shows that the scientific rigor of the RCT can open space for radical innovation. Because other scientific studies have shown a clear connection between bilingualism and cognitive development in many different contexts, because we know the relationship between nutrition and development, and because we understand how social networks combat toxic stress, not every project evaluation must prove exactly those hypotheses again and again.

Indigenous people on the Rio Negro explain that for some things, they need to look downstream, toward the knowledge and technology that comes from São Paulo or Europe; in other moments, they have to look (or travel) upstream to the more traditional villages where their ancestors lived for generations. We suggest a similar reflection for evaluations: in some moments, we need the rigor of an RCT to re-think and challenge our programs, but in other moments, we need to look upstream.

## Data Availability Statement

The raw data supporting the conclusions of this article will be made available by the authors, without undue reservation.

## Ethics Statement

The studies involving human participants were reviewed and approved by CONEP (Conselho nacional de ética em pesquisa), Brazil. The patients/participants provided their written informed consent to participate in this study.

## Author Contributions

All authors listed have made a substantial, direct and intellectual contribution to the work, and approved it for publication.

## Conflict of Interest

The authors declare that the research was conducted in the absence of any commercial or financial relationships that could be construed as a potential conflict of interest.

## Footnotes

^1^CanalCanoa and this research paper were financed by Saving Brains, an international funding consortium for early childhood, with funds from Grand Challenges Canada and the Fundação Maria Cecília Souto Vidigal.

^2^Hammer's self-criticism details how this happens: “There seems to be an unholy alliance between self-serving politicians and us researchers. They want to give out private goods since the recipient knows to whom to be grateful. We want to give them out since we can more easily separate treatment from control. Neither is helping the economy work better for everyone.”

^3^Ajuri is a Nheengatu word meaning “collective effort.”

^4^In only one ajuri were participants were not interviewed: a group of Hup'dah schoolteachers who live two weeks upriver, but were in town for a training session and requested an ajuri from CanalCanoa; interviewing participants after their return upriver was not logistically feasible. The research also excludes data from Ajuris done in indigenous land in collaboration with the Pastoral da Criança.

^5^The Nadehup and Yanomami forms of childrearing differ dramatically from the other ethnic groups in the region, and were as such not included in the films. Work with each group would require a new set of reference films.

^6^see also (17)

^7^During the course of the project, CanalCanoa staff worked to elaborate development milestones based on local values and skills; though this work opened space for a critique of other evaluation systems, it would demand much more work to be a rigorous system of evaluation or to serve as metrics for a randomized, controlled trial. CanalCanoa originally planned to work with every ethnic group in the region; however, the coordinators soon discovered that the riverine peoples had dramatically different childrearing techniques from the jungle people (Yanomami and Nadehup); no single series of films could encompass that diversity. In the same way, one would need to establish development milestones for radically diverse groups.

^8^See particularly the interview with the vice-secretary of health for the city of São Gabriel, Juarez Silva, in the film “Primeira Infância Indígena: Políticas Pública,” developed as a part of CanalCanoa. https://vimeo.com/357015971.
